# Stainless steel bipolar plate coated with polyaniline/Zn-Porphyrin composites coatings for proton exchange membrane fuel cell

**DOI:** 10.1038/s41598-020-60288-9

**Published:** 2020-02-24

**Authors:** M. A. Deyab, G. Mele

**Affiliations:** 1 Egyptian Petroleum Research Institute (EPRI), PO Box 11727, Nasr City, Cairo Egypt; 2 Department of Engineering for Innovation of University of Salento, via Arnesano, 73100 Lecce, Italy

**Keywords:** Electrochemistry, Energy

## Abstract

The proton exchange membrane fuel cells are the promising sustainable energy sources. The present study focuses on the enhancement the fuel cell performance and the protection of the stainless steel bipolar plate from the corrosion using polyaniline/Zn-Porphyrin composites coatings. The electrochemical properties (polarization and impedance) of the coated 303 stainless steel in 1.0 M H_2_SO_4_ solution have been evaluated. The coated 303 stainless steel by new composites exhibits the excellent anti-corrosion activity towards corrosive fuel cell electrolyte. The polyaniline/Zn-Porphyrin composite gives an excellent performance by adding 1.0% of Zn-Porphyrin. This composite improves the output power of the fuel cell.

## Introduction

Proton-exchange membrane fuel cells (PEMFC) create power by changing over chemical energy (hydrogen and oxygen gas) to electrical power. Because of the high cost of PEMFC components, the usage of this cell is limited^1^. The bipolar plates are the one of the main components in fuel cell. It works as conductor for electrical current from cell to cell^2^. The graphite is the foremost commonly utilized material for bipolar plates manufacture^3^. It has many advantages such as the great corrosion resistance. On another hand, there are many problems facing the use of graphite as the bipolar plates such as its brittle texture and high gas permeability^4^. The use of metallic materials for constructing bipolar plates has been highly welcomed in scientific circles^5–7^. They characterize by high electrical conductivity and low cost. Most commercialized bipolar plates made nowadays are stainless steel^8^. Metal corrosion is a huge problem, particularly in bipolar plates^9^. The presence of corrosion products and passive layer on the bipolar plate’s surface decrease the performance of PEMFC. To address this problem, many scientists have developed different conductive coatings to screen the metallic bipolar plates^10–12^. These coatings prevent the bipolar plate’s corrosion and consequently improve the PEMFC performance. Researchers have taken a major step towards protect the bipolar plates using conductive polymer coatings such as polyaniline (PANI)^13^. This kind of polymers is characterized by good conductivity and high thermal stability^14^. To maximize the efficiency of PANI, the combination of carbon nanotubes (CNT) with PANI was developed by many researchers^15^. Ramezanzadeh^16^ proposed polyaniline modified GO nanosheets coatings to improve the performance of stainless steel bipolar plate. Sharma and his colleagues^17^ improved the corrosion Resistance stainless steel bipolar plates using composite PANI and titanium nitride nanoparticle. Jiang *et al*.^18^ investigated the graphene oxide incorporated polypyrrole(PPY) matrix. The results showed that PPY-GO composite coatings work as good anti-corrosion coatings for stainless steel bipolar plates in the aggressive solutions. Gao *et al*.^19^ reported phosphomolybdic acid doped PANI coating for corrosion protection of 303SS. Show and his colleagues^20^ used CNT/PTFE composite coating for stainless steel bipolar plate. This coating decreased the contact resistance and increased the output power of the fuel cell. Here, we prepared a new composite coating for stainless steel bipolar plate. The base of this composite coating is polyaniline polymer (PANI) with Zn-Porphyrin (Zn-Pr). The main functions of new composite are the increasing the corrosion resistance of stainless steel bipolar plate and the enhancing output power of the fuel cell. The porphyrin molecules have very attractive properties. Its structure defined as a group of heterocyclic macrocycle organic compounds^21^. The insert of metals like Zn, Ni, and Co into porphyrin macrocycle structure influence on the optical absorption spectrum and the electrical and magnetic properties^21^. To our knowledge, this is the first study to use PANI/Zn-Pr composites coatings for PEMFC.

## Experimental

### Materials

Grade 303 stainless steel (303SS) (composition %: 0.15 C; 2.0 Mn; 1.0 Si; 0.2 P; 0.15 S; 17 Cr; 8 Ni; balance Fe) was used as the bipolar plate. The 303SS was cut into rectangle shape specimens with total surface area 1.12 cm^2^. These specimens were cleaned according to standard methods ASTM G1–03.

Polyaniline polymer and sulfuric acid (98%) were supplied from SigmaeAldrich Co. Xylene was supplied from PRABHAT CHEMICALS Co.

**Figure 1:**
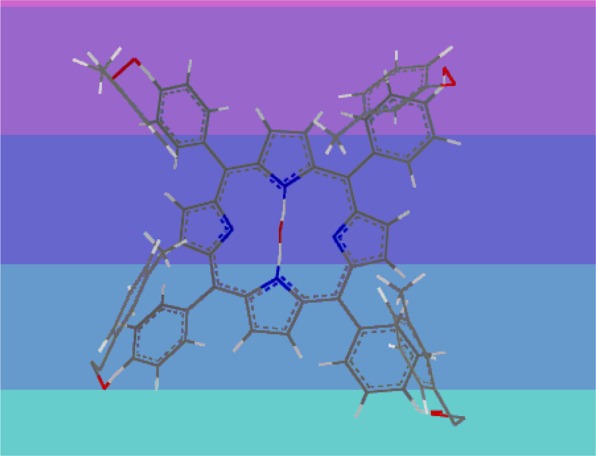
Zn-Porphyrin structure.

Zn-Porphyrin (Fig. 1) was synthesized according to the reported procedure^22^.

### PANI/Zn-Pr composites preparation and application of coating

The xylene (10 ml) and Zn-Pr powder (0.5, 0.8 and 1.0 gm) were mixed using mechanical stirrer (part 1). The xylene and PANI (1:1 ratio) (90 ml) were mixed using a high speed mechanical stirrer (part 2). The final composite was obtained by mixing part 1 and part 2 using mechanical stirrer followed by ultrasonication (3.0 h) and then ground for 1.0 h to obtain the desired fineness. PANI/Zn-Pr composites coatings were applied on the whole surface of clean 303SS using spray gun (Walther PILOT). The coated 303SS samples were cured at 343 K for 1.0 h.

The dry thickness of the PANI and PANI/Zn-Pr composites films was in the range 53 ± 5 μm using Elcometer 456 gauge (Elcometer Co).

Permeability Testing Cups (BYK Instruments) offer a simple method to check the permeability of PANI/Zn-Pr composites coatings within a 24 h period.

### Electrochemical measurements

The anti-corrosion of new PANI/Zn-Pr composite coating was evaluated using potentiodynamic polarization and electrochemical impedance spectroscopy (EIS) techniques.

The polarization curves were recorded using recommended scan rate (1.25 mV s^−1^) in the potential range ± 250 mV with regard to the open circuit potential.

EIS plots were recoded in the the frequency range 0.01 Hz–100 kHz at the open circuit potential. The AC voltage amplitude was 10 mV. Electrochemical data were collected using Potentiostat/Galvanostat (model Gill AC, 947, ACM).

The electrochemical experiments were repeated at least 3 times to ensure accuracy. All values are presented in the form of mean values and standard deviation.

### PEMFC performance measurements

For the PEMFC performance test, the laboratory single cell fuel cell testing stations from Fuel Cell Store was used. In this cell, H_2_ and O_2_ gas were flow with rate 150 ml/min. Bare 303SS and coated 303SS were used as bipolar plates. Nafion 117 was used as the proton electrolyte membrane. Platinum particles on acetylene black powder (as support) were used as the catalyst on the surface of anode and cathode electrodes.

## Results and Discussion

### Anti-corrosion performance of PANI/Zn-Pr composites

**Figure 2:**
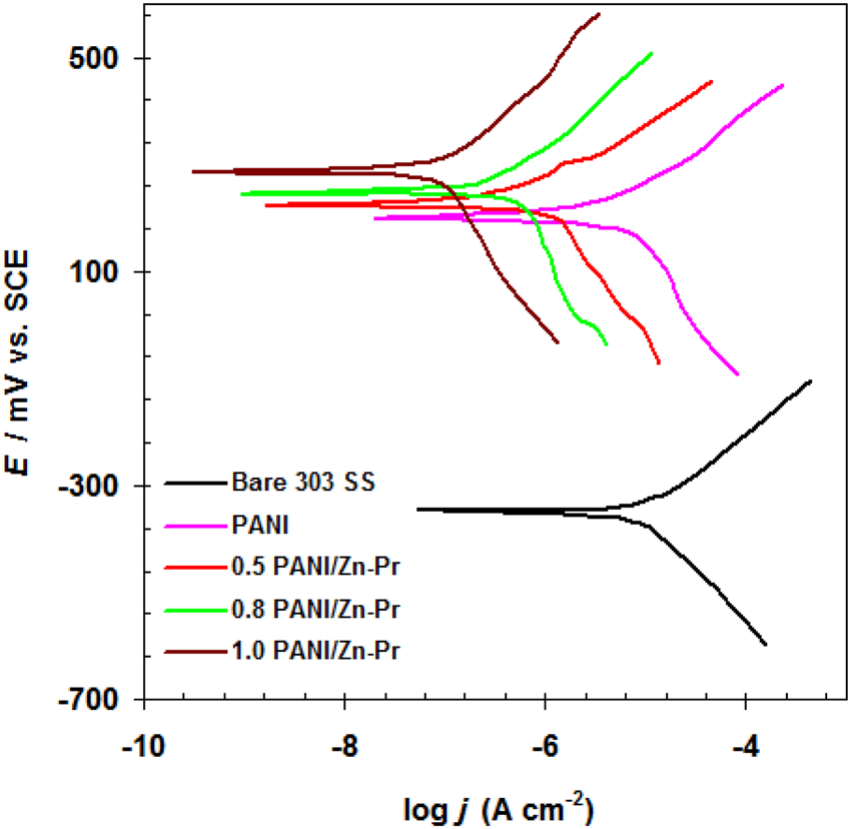
Polarization curves of uncoated and coated 303SS by neat PANI and PANI/Zn-Pr composites in 1.0 M H_2_SO_4_ solution at 298 K.

We first recoded the polarization responses of uncoated stainless steel bipolar plate 303SS in 1.0 M H_2_SO_4_ solution. The same experiments were applied for coated 303SS by neat PANI and PANI/Zn-Pr composites. All these curves are collected in Fig. 2.

The corrosion potential (*E*_corr_) and corrosion current density (*j*_corr_) were extracted form Tafel curves^23^ (see Table 1) to assess anti-corrosion performance of PANI/Zn-Pr composites.

**Table 1:** Electrochemical parameters and the corresponding corrosion inhibition efficiency for uncoated and coated 303 SS in 1.0 M H_2_SO_4_ solution at 298 K.

Sample	*j*_corr_ (μA cm^−2^)	*E*_corr_ mV vs. SCE	*η*_j_ %
Bare 303SS	25.68 ± 1.05	−347 ± 5.7	—
PANI	5.90 ± 0.45	193 ± 3.8	77.02
0.5% PANI/Zn-Pr	1.24 ± 0.23	221 ± 4.3	95.17
0.8% PANI/Zn-Pr	0.39 ± 0.04	224 ± 4.8	98.48
1.0% PANI/Zn-Pr	0.15 ± 0.02	284 ± 5.1	99.41

The *j*_corr_ values of coated 303SS by neat PANI and PANI/Zn-Pr composites were much lowers than that of the control 303SS. The incorporation of Zn-Pr molecules into PANI leads to increase its anti-corrosion performance. This is clearly shown through the considerable decrease of *j*_corr_ values form 5.90 μA cm^−2^ to 0.15 μA cm^−2^. It was noted also there is a significant shift in *E*_corr_ to more noble direction in the cases of PANI/Zn-Pr composites, indicating the protective effect of new composites^24^. Here, the composites layer covered both cathodic and anodic sites on the surface of 303 SS and caused the shifting in the *E*_corr_ values.

We calculated anti-corrosion efficiencies (*η*_j_%) of coatings from the Eq. (1)^25^.

1 $${{\eta }}_{j} \% =\frac{{{j}}_{{\rm{corr}}}^{o}-{{j}}_{{\rm{corr}}}^{c}}{{{j}}_{{\rm{corr}}}^{o}}\times 100$$

where *j*^*o*^_corr_ and *j*^*c*^_corr_ are the corrosion rates for uncoated and coated 303 SS, respectively.

In the case of coated samples with neat PANI, the *η*_j_% was 77.02% (see Table 1). The use of PANI/Zn-Pr composites leads to enhance the anti-corrosion efficiency of PANI. The maximum efficiency was obtained at 1.0% of Zn-Pr (Table 1).

The permeability of composites coatings is an important property to measure the electrolyte transfer through a coatings film. The presence of pores across the PANI coating is responsible for allowing corrosive cell electrolyte permeation. Therefore, blocking these pores can result in effectively hindering the corrosion of bipolar plates. In this respect, the dispersion of Zn-Pr particles in the texture of PANI coating leads to low electrolyte permeability of the PANI/Zn-Pr composites compared to neat PANI coating (see Table 2).

**Table 2:** Water permeability of PANI coating in the absence and presence of Zn-Pr at 298 K.

Sample	H_2_O permeability cm^2^ s^−1^
PANI	1.36 × 10^−6^
0.5% PANI/Zn-Pr	2.55 × 10^−7^
1.0% PANI/Zn-Pr	9.03 × 10^−8^

### Electrochemical impedance spectroscopy

To exclude the effect of anti-corrosion and conductivity activities caused by the addition of Zn-Pr, we measured the electrochemical impedance responses (Nyquist plot) of bare 303SS, neat PANI and PANI/Zn-Pr composites in 1.0 M H_2_SO_4_ solution. All measurements were achieved after 7 days.

**Figure 3:**
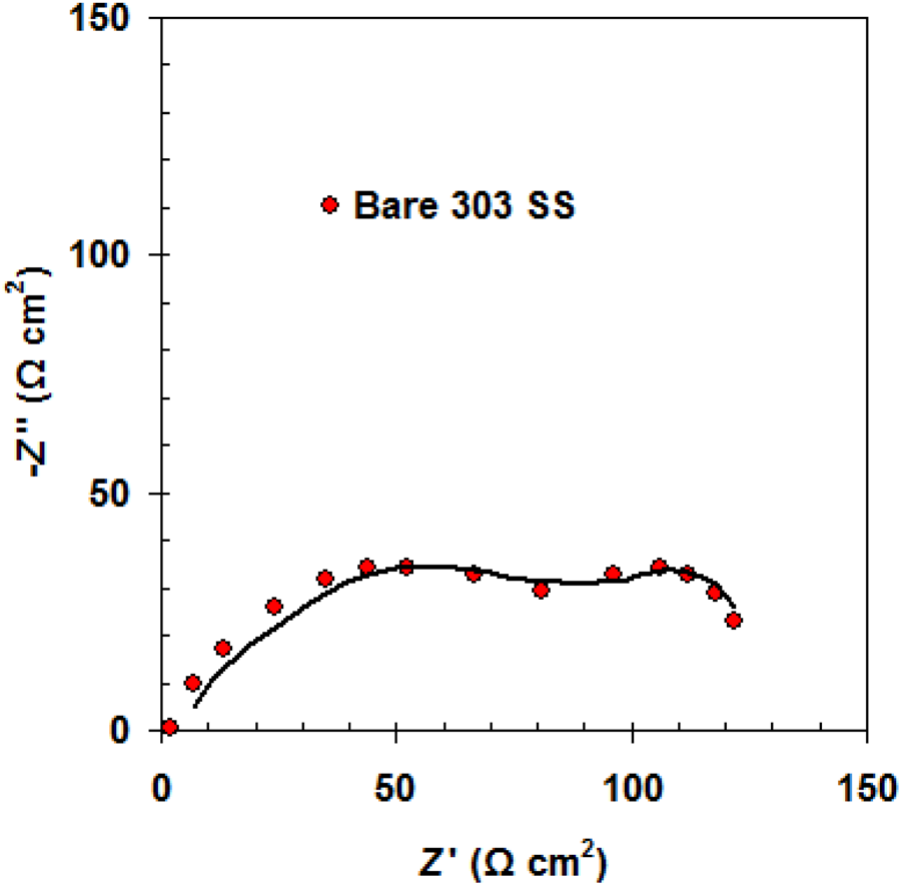
Nyquist plot of bare 303 SS in 1.0 M H_2_SO_4_ solution at 298 K.

**Figure 4:**
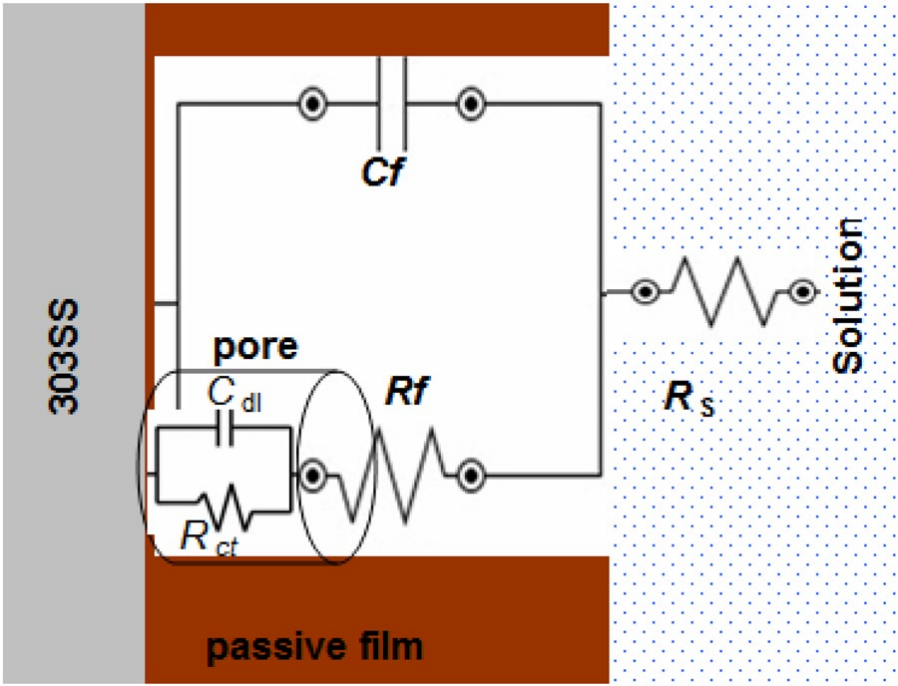
Equivalent circuit to fit the impedance bare 303 SS in 1.0 M H_2_SO_4_ solution.

We found that the Nyquist plots for bare 303SS comprise of two capacitive loops (see Fig. 3). The best an equivalent circuit described this case is given in Fig. 4.

**Figure 5:**
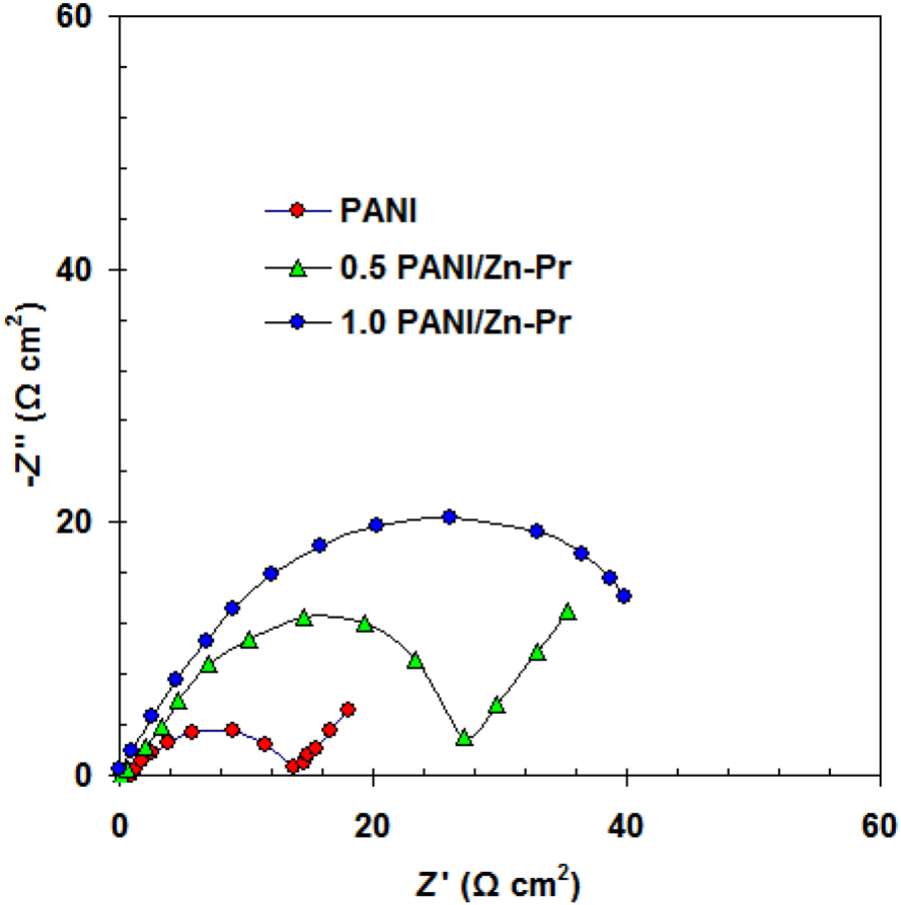
Nyquist plots for coated 303 SS by the neat PANI and PANI/Zn-Pr composites in 1.0 M H_2_SO_4_ solution at 298 K.

**Figure 6:**
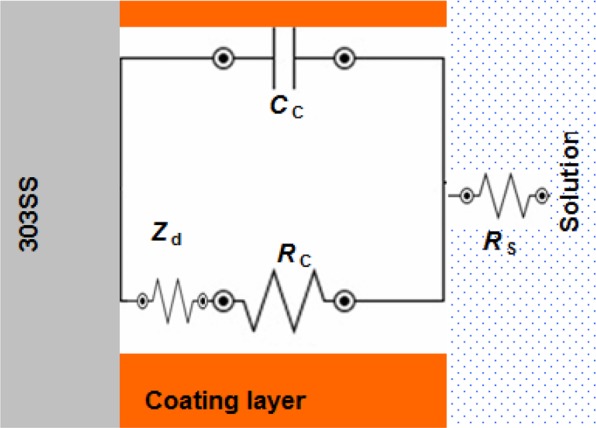
Equivalent circuit to fit the impedance coated 303 SS by the neat PANI and PANI/Zn-Pr composite (0.5% Zn-Pr) in 1.0 M H_2_SO_4_ solution.

The electrolyte resistance was represented by *R*_s_. The passive film resistance and capacitance were represented by *R*_f_ and *C*_f_, respectively. *R*_ct_ and *C*_dl_ are charge transfer resistance and double layer capacitor related to 303SS/H_2_SO_4_ solution interface^26^. We noted that the Nyquist plots for coated samples by the neat PANI and PANI/Zn-Pr composites were changed to one capacitive loop (at high frequencies) and straight line (at low frequencies) (Fig. 5)^27^. This line is due to diffusion impedance of the coating barrier layer (*Z*_d_)^28^. The equivalent circuit described, the PANI and PANI/Zn-Pr composites (0.5% Zn-Pr) is shown in Fig. 6. In this case, *C*_c_ and *R*_c_ represent the capacitance and resistance of the coating layer, respectively.

**Figure 7:**
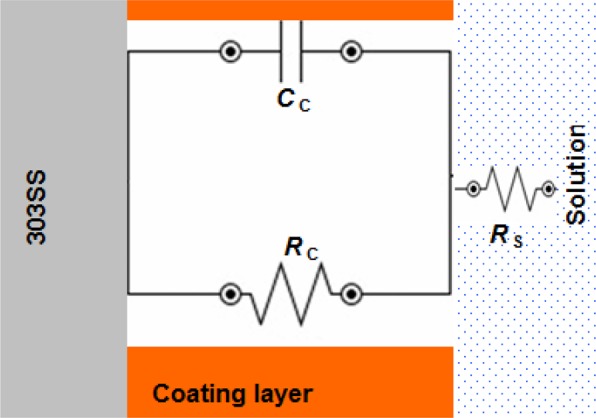
Equivalent circuit to fit the impedance coated 303 SS by PANI/Zn-Pr composite (1.0% Zn-Pr) in 1.0 M H_2_SO_4_ solution.

By incorporation 1.0% of Zn-Pr into PANI coating, the *Z*_d_ part for diffusion process was disappeared and the equivalent circuit was represented only by *C*_c_ and *R*_c_ elements (see Fig. 7).

All the equivalent circuit elements for uncoated and coated samples are listed in Tables 3 and 4.

**Table 3:** Fitted electrochemical parameters of 303 SS in 1.0 M H_2_SO_4_ solution at 298 K.

Sample	*R*_f_ Ω cm^2^	*C*_f_ μF cm^−2^	*R*_ct_ Ω cm^2^	*C*_dl_ μF cm^−2^
Bare 303 SS	76.40 ± 2.3	204 ± 4.7	55.65 ± 1.3	193 ± 2.6

**Table 4:** Fitted electrochemical parameters of coated 303 SS in 1.0 M H_2_SO_4_ solution at 298 K.

Sample	*R*_c_ Ω cm^2^	*C*_c_ μF cm^−2^	*Z*_d_ Ω cm^2^
PANI	13.6 ± 1.2	398 ± 3.5	6.8 ± 0.3
0.5% PANI/Zn-Pr	26.8 ± 1.3	234 ± 4.6	14.6 ± 0.4
1.0% PANI/Zn-Pr	48.3 ± 1.6	212 ± 2.7	—

Initially the *R*_c_ value of PANI coatings is comparatively low than for passive film *R*_f_. This implies that the PANI coatings have considerable conductivity if it compares with the passive layer formed on the bare 303SS^18,29^. By incorporation Zn-Pr into PANI coating, the coating resistance *R*_c_ increased and the coating capacitance *C*_C_ decreased.

Here, Zn-Pr particles repair the PANI coating flaws and block the passage of corrosive solution towards 303 SS substrate and caused the changing in the *R*_c_ and *C*_C_ values.

This indicates that Zn-Pr plays a vital role in enhancing the anti-corrosion property of PANI coatings. Furthermore, at 1.0% of Zn-Pr, the PANI coating become a very good barrier for restriction the diffusion of corrosive solution.

### PEMFC performance

**Figure 8:**
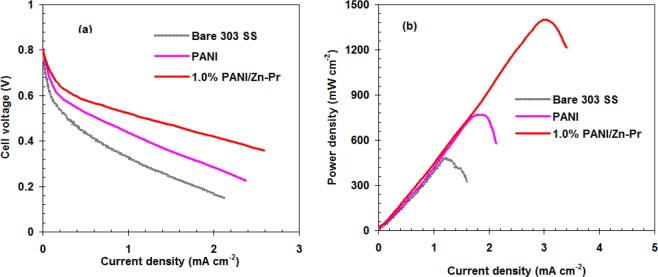
Output cell voltage (**a**) and power density (**b**) for single PEMFC cell using uncoated and coated 303 SS bipolar plates.

To assess the effect of the PANI/Zn-Pr composites coating on the PEMFC Performance, we explored the reliance of the current density on the output cell voltage and the power density for single PEMFC cell using uncoated or coated 303SS bipolar plates. This data was showed in Fig. 8(a,b).

The open voltage for all samples was 0.805 V. Generally, the out voltage cell decreased with current density increasing due to IR drop^30^. The out voltage in the case of coated 303SS bipolar plates by PANI/Zn-Pr composites was significantly decreased comparing with bare bipolar plates and neat PANI bipolar plates (see Fig. 8(a)).

The supreme power density for single PEMFC cell using uncoated 303SS bipolar plates is 435 mW cm^−2^ (Fig. 8(b)). This value was increased to 720 and 1353 mW cm^−2^ in the case of neat PANI and PANI/Zn-Pr composite, respectively. This confirms that the using of PANI-Zn-Pr composite leads to increase the performance of PEMFC cell.

### Mechanism and explanation

The above results reveal the strong effect of Zn-Pr on the anti-corrosion effect of PANI coating for 303SS bipolar plates, leading to an increased PEMFC performance.

The 303SS bipolar plates suffered from the corrosion and dissolution during the immersion in cell electrolyte. In addition, the passive film formed during the corrosion process, led to the increase in the contact resistance and the membrane electrodes contamination^31,32^.

The PANI coating plays as a barrier for 303SS bipolar plates. Where this coating decreases the contact between the 303SS surface and corrosive electrolyte. But the performance of PANI coating is not high enough to prevent the bipolar plate’s corrosion due to the presence of pores in the texture of PANI polymer^33^. These pores allow the passage of corrosive solution to contact with the bipolar plates^34^. The corrosive ions enhance the active dissolution of 303SS^35,36^. These ions may lead to pitting corrosion and induce metal failure^37^.

The incorporation of Zn-Pr with PANI polymer to form PANI/Zn-Pr composite, leads to efficiency coating barrier. The main role of Zn-Pr is the reducing the pores in the PANI polymer texture^10,38^. This will significantly affect on the degree of contact between the 303SS surface and corrosive electrolyte.

Additionally, the presence of Zn-Pr molecules act as a conductive path though PANI polymer, leading to the increase in the output power of the PEMFC cell^39,40^. The conducting characterization of Zn-Pr molecules is due to the aromaticity of the macrocycle, leading to the electrons mobility though the PANI/Zn-Pr composite^41,42^.

## Conclusions

PANI/Zn-Pr composites coatings have been developed, which is effective to significantly increase the output power of the PEMFC cell and decrease the degree of contact between the 303SS bipolar plates and corrosive electrolyte. Anti-corrosion properties of PANI/Zn-Pr composites were confirmed using polarization experiments to define their anti-corrosion efficiency. The highest anti-corrosion activity of the PANI/Zn-Pr composite (i.e. 99.41%) was obtained at 1.0% of Zn-Pr. EIS data also further confirmed that the Zn-Pr plays a significant role in enhancing the coating resistance of PANI. The use of coated 303SS bipolar plates by PANI/Zn-Pr composites leads to increase the output density for single PEMFC cell. Therefore, the new PANI/Zn-Pr composites are considered effective coatings for bipolar plates in future PEMFC technologies.
